# Supplementary figures and images for: Star Power: Early life stages of an endangered sea star are robust to current and near-future warming
Source: PLoS One. 2025 Sep 3;20(9):e0318879. doi: 10.1371/journal.pone.0318879 (PMC12407436; doi:10.1371/journal.pone.0318879)

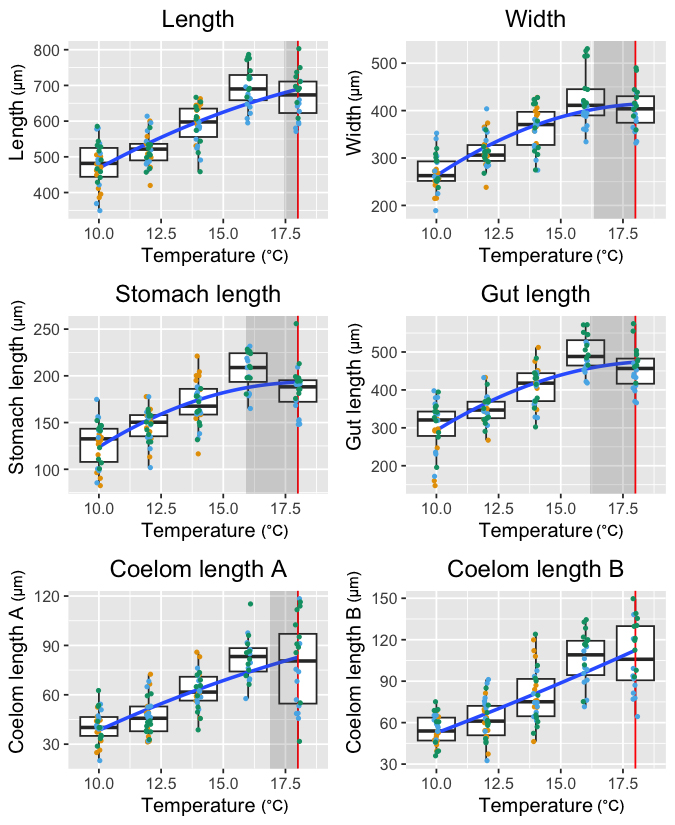

Supplement: S1 Fig — Raw larval character measurements in the embryo across different temperature exposures, Exp 1. Individual points represent individual larvae, with different colors for each replicate. Blue lines show the quadratic fits. Vertical red lines show the estimates for optimal temperature for each feature; the shaded regions indicate the estimated 95% confidence intervals. See S1 Table for description of characters. (JPG) [file pone.0318879.s001.jpg]

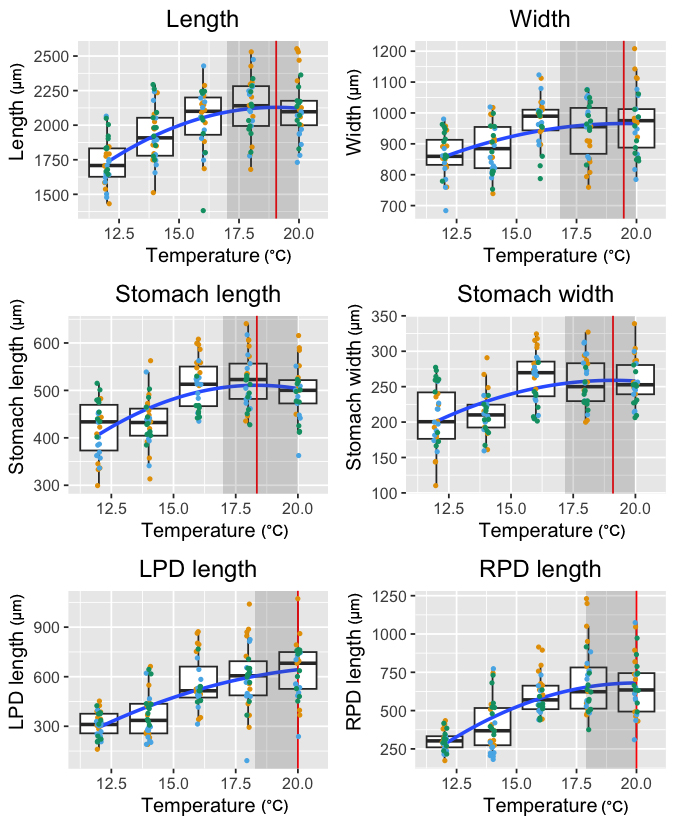

Supplement: S2 Fig — Raw larval character measurements in the bipinnaria (mid) stage larva across different temperature exposures, Exp 1. Individual points represent individual larvae, with different colors for each replicate. Blue lines show the quadratic fits. Vertical red lines show the estimates for optimal temperature for each feature; the shaded regions indicate the estimated 95% confidence intervals. See S1 Table for description of characters. (JPG) [file pone.0318879.s002.jpg]

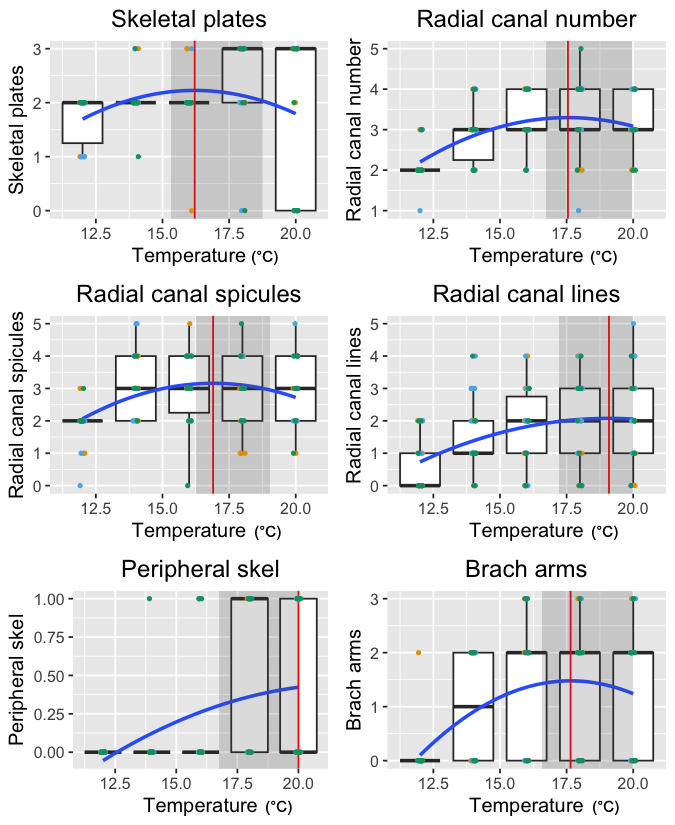

Supplement: S3 Fig — Raw juvenile character measurements in the bipinnaria (mid) stage larva across different temperature exposures, Exp 1. Individual points represent individual larvae, with different colors for each replicate. Blue lines show the quadratic fits. Vertical red lines show the estimates for optimal temperature for each feature; the shaded regions indicate the estimated 95% confidence intervals. See S1 Table for description of characters and units. (JPG) [file pone.0318879.s003.jpg]

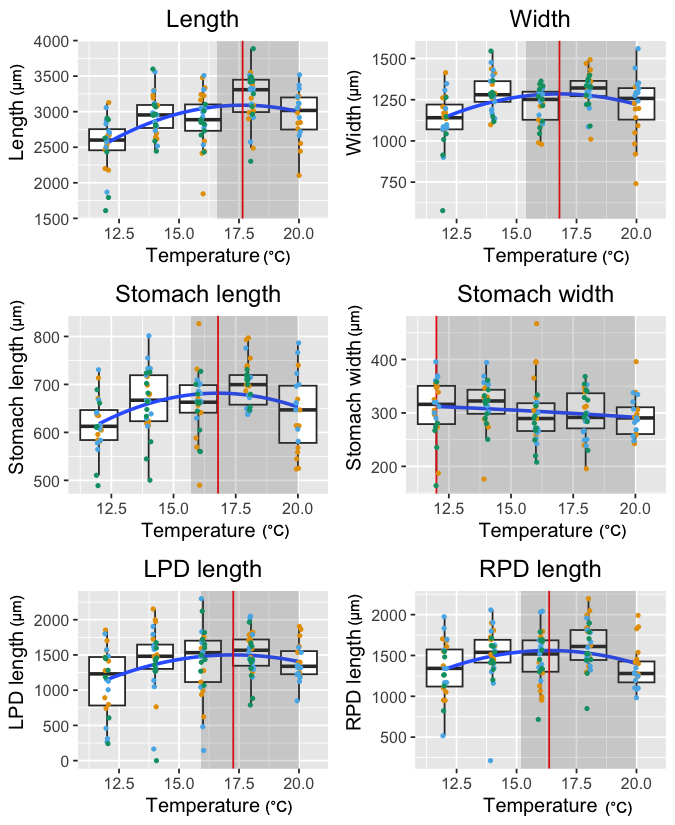

Supplement: S4 Fig — Raw larval character measurements in the brachiolaria (late) stage larva across different temperature exposures, Exp 1. Individual points represent individual larvae, with different colors for each replicate. Blue lines show the quadratic fits. Vertical red lines show the estimates for optimal temperature for each feature; the shaded regions indicate the estimated 95% confidence intervals. See S1 Table for description of characters. (JPG) [file pone.0318879.s004.jpg]

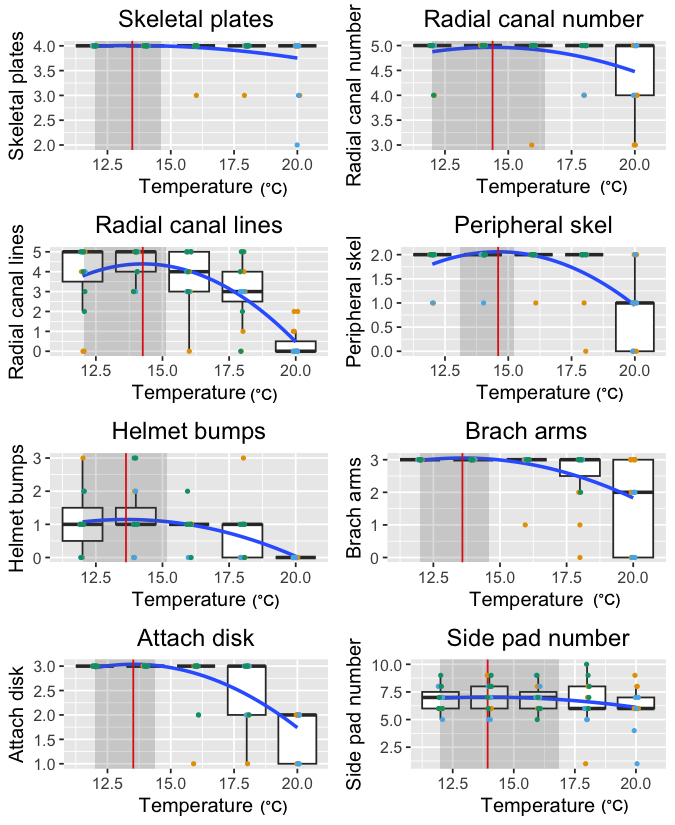

Supplement: S5 Fig — Raw juvenile character measurements in the brachiolaria (late) stage larva across different temperature exposures, Exp 1. Individual points represent individual larvae, with different colors for each replicate. Blue lines show the quadratic fits. Vertical red lines show the estimates for optimal temperature for each feature; the shaded regions indicate the estimated 95% confidence intervals. See S1 Table for description of characters and units. (JPG) [file pone.0318879.s005.jpg]

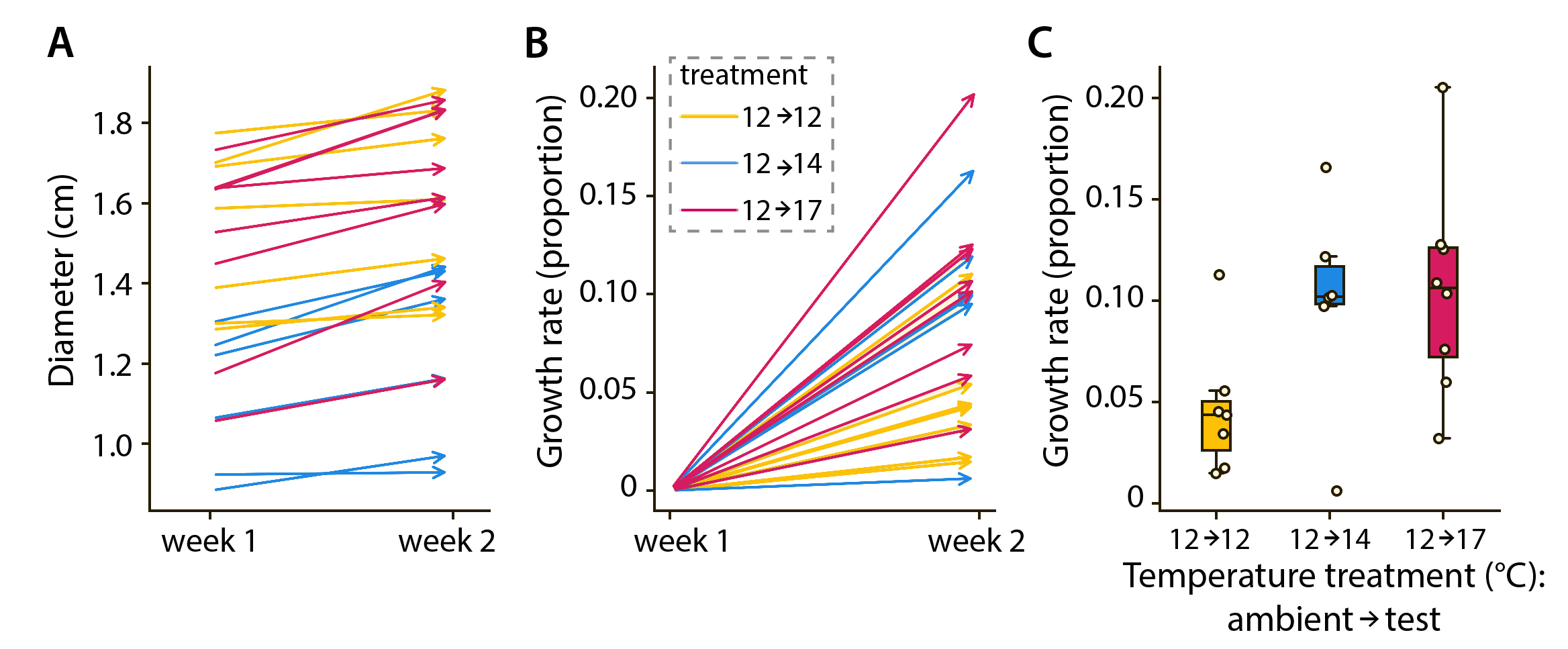

Supplement: S6 Fig — (A) Week 1 shows the diameter of each star recorded during the ambient flip test, Week 2 shows the diameter of the same star one week later during the experimental flip test. Yellow– stars from 12- > 12°C treatment; blue– 12- > 14.5°C treatment; pink– 12- > 17°C treatment. (B) Same data as in A but expressed as growth rate: (Diam2-Diam1)/Diam1. (C) Standard box plot of the growth rate data from B. Stars in all three treatments grew measurably (p < 0.05). Although we detected no significant differences among treatments in growth rate by standard criteria (p < 0.05), there was a hint of faster growth at 12- > 17°C versus 12- > 12°C (p = 0.066; see the text). (JPG) [file pone.0318879.s006.jpg]
