# Supplementary material for: Star Power: Early life stages of an endangered sea star are robust to current and near-future warming
Source: PLoS One. 2025 Sep 3;20(9):e0318879. doi: 10.1371/journal.pone.0318879 (PMC12407436; doi:10.1371/journal.pone.0318879)
Supplement: S1 Table — Fig references are either to panels in Fig 1 in this publication, or to panels in Figure 3 in Hodin et al 2021 (Biol Bull 241: 243–258). Stage measured: E–embryo; L1–mid larva (bipinnaria); L2-late larva (brachiolaria). (PDF) [file pone.0318879.s007.pdf]

S1 Table. List and description of characters measured or scored in *Exp 1*. Fig references are either to panels in Fig 1 in this publication, or to panels in Figure 3 in Hodin et al 2021 (*Biol Bull* 241: 243-258). Stage measured: E–embryo; L1–mid larva (bipinnaria); L2–late larva (brachiolaria).

Character	Stage measured	Larv or Juv character?	Description	Fig reference
Length	E, L1, L2	Larval	Anterior-posterior (A-P) length measured through midline.	Fig 1A,B
Width	E, L1, L2	Larval	Width measured perpendicular to and at mid-point of length.	See Fig 1 legend
Stomach length	E, L1, L2	Larval	A-P length measured through midline of stomach along the gut axis.	Fig 1B
Stomach width	L1, L2	Larval	Width measured perpendicular to and at mid-point of stomach length.	See Fig 1 legend
Gut (or invagination) length	E	Larval	Invagination measured from outer edge of ectoderm to posterior most edge of invagination; in complete gut, measured from anterior lip of mouth to posterior edge of stomach.	Fig 1A
Coelom length (A,B)	E	Larval	Maximum length parallel to A-P axis of embryo; measured left (L) & right (R) coeloms but did not distinguish side. “Coelom A” was the smaller of the two coeloms in each embryo; “Coelom B” was the larger.	Fig 1A
Posterodor-sal (RPD, LPD) arm length	L1, L2	Larval	Traced along posterior edge of arm out to arm tip; distinguished L & R arms.	Fig 1B
Skeletal plates	L1, L2	Juvenile	Most advanced plate visible: 0–none; 1–wishbone (WB); 2–WB+; 3–WB→ Snowflake (SF); 4–SF. WB: linear spicule with bifurcations at each end; WB+: at least one additional bifurcation; WB→SF: plate forming with at least one ‘cell’ (enclosed ring) visible; SF: 4 or more cells visible in at least one plate.	Fig 1D<br>Fig 3A,C-E in Hodin et al 2021
Peripheral skeleton	L1, L2	Juvenile	Stage of spine formation at posterior periphery (stages 1-3 shown L→R at right): 0–none; 1–spicule dot; 2–spicule; 3–spine.	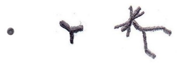<br>Fig 3H,I in Hodin et al 2021

Radial canal number	L1, L2	Juvenile	Number of radial canals visible on left side of stomach (0-5).	Fig 1B,D
Radial canal (skeletal) spicules	L1	Juvenile	Number of radial canals (0-5) that have at least one skeletal spicule.	Fig 1D
Radial canal (skeletal) lines	L1, L2	Juvenile	Number of radial canals (0-5) that have at least one linear spicule (more advanced stage).	Fig 3D in Hodin et al 2021
“Helmet” bumps	L2	Juvenile	Number of prominent protrusions visible at posterior.	Fig 3G in Hodin et al 2021
Brachiolar (Brach) arms	L1, L2	Juvenile	0–None; 1–brachiolar buds; 2–brachiolar arms (can move) but without ‘bumpy’ tips; 3–brachiolar arms with bumpy tips (mature).	buds: Fig 1C<br>arms: Fig 1B<br>mature: Fig 3J in Hodin et al 2021
Attachment (Attach) disk	L2	Juvenile	0–Disk absent; 1–diffuse (not forming a clean circle); 2–condensing but not birefringent (in cross polarized light); 3–mature and birefringent.	condensing: Fig 3F in Hodin et al 2021<br>mature: Fig 1C
Side pad number	L2	Juvenile	Number of accessory structures (‘side pads’) lateral to attachment disk visible in brachiolar complex (0-10).	Fig 1C<br>Fig 3F,J in Hodin et al 2021
