# Supplementary material for: Star Power: Early life stages of an endangered sea star are robust to current and near-future warming
Source: PLoS One. 2025 Sep 3;20(9):e0318879. doi: 10.1371/journal.pone.0318879 (PMC12407436; doi:10.1371/journal.pone.0318879)
Supplement: S2 Table — Weights assigned to individual characters (see S1 Table) for constructing the larval or juvenile PC1 composite variable in Exp 1, which models larval or juvenile development at each stage. Dashes indicate characters that were not analyzed at that stage. (PDF) [file pone.0318879.s008.pdf]

S2 Table. Weights assigned to individual characters (see S1 Table) for constructing the larval or juvenile PC1 composite variable in *Exp 1*, which models larval or juvenile development at each stage. *Dashes* indicate characters that were not analyzed at that stage.

	**Embryo**	**Bipinnaria**	**Brachiolaria**
**Larval features**			
Length	0.44	0.41	0.5
Width	0.43	0.37	0.49
Gut length	0.44	-	-
Coelom A length	0.33	-	-
Coelom B length	0.38	-	-
Stomach length	0.41	0.45	0.45
Stomach width	-	0.39	0.23
Left posterolateral (LPL) arm	-	0.41	0.35
RPL arm	-	0.41	0.36
**Juvenile features**			
Skeletal plates	-	-0.02	0.34
Radial canal number	-	0.5	0.32
Radial canal spicules	-	0.52	-
Radial canal lines	-	0.5	0.39
Peripheral spines	-	0.28	0.41
Brach arms	-	0.39	0.38
Helmet bumps	-	-	0.27
Attach disk	-	-	0.43
Side pad number	-	-	0.25
