# Supplementary material for: Star Power: Early life stages of an endangered sea star are robust to current and near-future warming
Source: PLoS One. 2025 Sep 3;20(9):e0318879. doi: 10.1371/journal.pone.0318879 (PMC12407436; doi:10.1371/journal.pone.0318879)
Supplement: S3 Table — Rates of cloning were higher at 20°C when compared to the other temperature treatments (Z3,15 = 3.134; p < 0.01), though this effect was largely driven by 20°C Replicate C. The four classes of larvae are further described in the Methods. (PDF) [file pone.0318879.s009.pdf]

S3 Table. Larval cloning in *Exp 1* at 64 dpf. Rates of cloning were higher at 20°C when compared to the other temperature treatments ($Z_{3,15}$=3.134; p<0.01), though this effect was largely driven by 20°C Replicate C. The four classes of larvae are further described in the Methods.

**Treatment (°C)**	**Replicate**	**Class 1 larvae** (full size)	**Class 2 larvae** (regenerating clones)	**Class 3 larvae** (small clones)	**Class 4 larvae** (tiny clones)
12	A	48	9	5	4
12	B	50	4	4	8
12	C	45	11	6	9
14	A	65	6	3	1
14	B	46	9	10	7
14	C	52	13	9	5
16	A	30	10	12	10
16	B	53	9	2	4
16	C	50	15	7	2
18	A	64	10	1	5
18	B	56	13	4	6
18	C	36	17	9	17
20	A	49	3	13	3
20	B	59	19	1	2
20	C	0	10	12	35
