# Supplementary material for: Star Power: Early life stages of an endangered sea star are robust to current and near-future warming
Source: PLoS One. 2025 Sep 3;20(9):e0318879. doi: 10.1371/journal.pone.0318879 (PMC12407436; doi:10.1371/journal.pone.0318879)
Supplement: S4 Table — Rates of cloning were 50% higher at 17°C when compared to either 11°C (Z3,9 = 7.518; p < 0.001) or 14°C (Z3,9 = 7.234; p < 0.001). (PDF) [file pone.0318879.s010.pdf]

S4 Table. Larval cloning in *Exp 3* at 63 dpf. Rates of cloning were 50% higher at 17°C when compared to either 11°C ($Z_{3,9}$=7.518; p<0.001) or 14°C ($Z_{3,9}$=7.234; p<0.001).

Treatment	Replicate	Clones	Fully-grown larvae
11°C	A	115	119
11°C	B	85	127
11°C	C	86	170
14°C	A	97	136
14°C	B	110	123
14°C	C	76	137
17°C	A	97	55
17°C	B	68	42
17°C	C	98	48
