# Supplementary material for: Star Power: Early life stages of an endangered sea star are robust to current and near-future warming
Source: PLoS One. 2025 Sep 3;20(9):e0318879. doi: 10.1371/journal.pone.0318879 (PMC12407436; doi:10.1371/journal.pone.0318879)
Supplement: Attachment — Submitted filename: response.pdf [file pone.0318879.s012.pdf]

Dear Dr. Fugmann

Thank you for your email giving us an opportunity to revise our manuscript: *Star Power: Early life stages of an endangered sea star are robust to current and near-future warming*.

We first reply to the points in the body of your message and then go on to address the two suggestions of Reviewer #1, Dr. JP Rast.

1. We believe we have now followed all style requirements as seen in the links you helpfully provided – if anything needs fixing, please let us know and we are happy to do so.

2. We started the process of formatting the submission with LaTex as requested. Because we have never used this formatting method, we have found it rather onerous. We are requesting guidance if this is indeed necessary, and if so we will require more time to reformat the file in LaTex. Here we provide a marked up word doc version (with changes tracked) for your convenience in reviewing our revisions.

3. We do not need to change the funding statement. The additional funding not found in the funding statement that we listed in the originally submitted Acknowledgements was our thanks to "dozens of donors." This was not "funding" per se as they were charitable gifts via our University with no stipulations on how the money is to be spent. Therefore we do not feel it should be mentioned in a formal Funding section. And we have removed it as requested (along with other mention of financial support) from the Acknowledgments,

4. We are currently preparing our data for upload to the referenced database. This should be done within a few weeks. We do not expect this to hold up the timeline. The permanent link for the data will be as follows:

DOI: 10.5061/dryad.msbcc2g9s

5. see point 4, above.

6. We have removed all "data not shown" references as requested. In one instance we now point to the raw uploaded data that supports our claim (see line 1112 in the marked up version; and please advise if this is properly stated). In one other we have added statistical support for a statements previously listed as data not shown. Others seemed to stand fine as is without a reference to data, shown or otherwise.

7. We have removed the sentence citing COSEWIC, and have taken the reference out of the reference list.

8. We believe the reference list is correct as now submitted.

Reviewer #1 (JP Rast)

Since the system under consideration consists of both larvae and their food algae, are temperature effects on the algae also something that may affect growth?

Or temperature effects of settlement substrate diatoms, coralline algae?

We thank Dr. Rast for his kind comments on the manuscript, and appreciate these questions. To address them we have added a paragraph to the Discussion (see lines 563-573 in the appended marked-up word doc version) discussing possible temperature effects on these additional organisms included, of necessity, in some of our trials. We also indicate why any such interaction effects would not impact our overall conclusions.
